# Midbrain dopaminergic neuron fate specification: Of mice and embryonic stem cells

**DOI:** 10.1186/1756-6606-1-8

**Published:** 2008-09-30

**Authors:** Emily Gale, Meng Li

**Affiliations:** 1 MRC Clinical Sciences Centre, Imperial College London, London, UK

## Abstract

The midbrain dopaminergic (mDA) neurons of the substantia nigra and the ventral tegmental area play a fundamental role in the control of voluntary movement and the regulation of emotion, and are severely affected in Parkinson's disease. Recent advances in mouse genetics and vertebrate development have provided us with insight into the genetic cascades involved in the development of mDA neurons, including the induction of mDA neuron progenitors in the ventral mesencephalon, the specification of the mDA neuronal fate and the maintenance of postmitotic mDA neurons. In parallel, rapid progress has been made in the generation of DA neurons from pluripotent stem cells and the development of stem cell-based therapies for Parkinson's disease. Here, we summarize the new findings via the developmental progression of mDA neurons and outline how this knowledge has been exploited to develop novel paradigms for the in vitro generation of these neurons from embryonic stem cells.

## Introduction

Dopamine containing neurons are present in different positions in the vertebrate central nervous system with the largest assembly in the midbrain. Midbrain dopaminergic (mDA) neurons are separated into functionally distinct subgroups called the substantia nigra compacta (SNc (also called the A9 group) and the ventral tegmental area (VTA (also called the A10 group) based on their position within the midbrain and the target structures which they innervate [1]. Dopaminergic neurons of the SNc primarily project to the dorsolateral striatum and regulate motor function. The VTA neurons, on the other hand, project to the ventromedial striatum, cortical areas and the limbic system and are involved in emotional behaviour and mechanisms of natural motivation and reward. In humans, the preferential degeneration of SNc neurons results in Parkinson's disease whilst defects of the VTA neuron system are implicated in psychiatric disorders.

Because of their involvement in Parkinson's disease and other mental disorders, mDA neurons have been a focus of clinical interest and a subject of intensive studies for a long time. For Parkinson's disease, a potential therapy is to replace the lost mDA neurons with healthy DA neurons that have been generated in vitro through the differentiation of stem cells. To achieve this, a comprehensive understanding of the genetic cues and extrinsic signalling cascade controlling the fate choice of pluripotent embryonic stem (ES) cells into neuroepithelial stem cells and subsequently into functional midbrain specific DA neurons is required. In this regard, recent studies have identified a number of regulatory factors that influence the emergence of mDA neurons during vertebrate embryogenesis. These studies not only have increased our understanding of mDA neuron development in vivo, they have also guided the development of new paradigms for the in vitro generation of mDA neurons from ES cells. In return, ES cell differentiation in vitro provides a powerful research tool for the genetic dissection of mDA neuron development and cell biology.

Several excellent reviews have extensively analyzed the extrinsic signalling pathways and genetic programme governing mDA neuronal differentiation and functional maturation [2-7]. In this article we summarise insights from recent studies of mDA neuron fate specification in animal models, highlighting the pivotal role of fundamental developmental studies in devising novel strategies harnessing ES cell differentiation to produce the mDA neuronal phenotype.

## From epiblast to midbrain DA neurons

**Figure 1:**
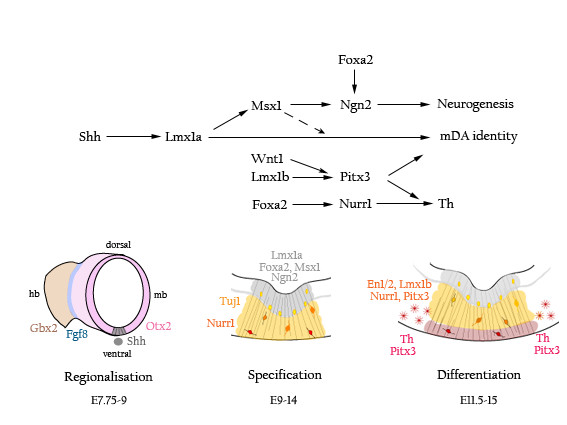
**Schematics of the key players of mDA neuron development.*** Regionalisation *of the neural tube (hindbrain (hb) brown, midbrain (mb) pink) establishes midbrain tissue identity via the inductive signals of Shh and Fgf8, which arise from the notochord (grey circle) and the midbrain-hindbrain border (blue) respectively, combined with Otx2 expression. This interaction enables midbrain ventral midline cells to respond to the later expression of the mDA neuron determination gene Lmx1a. *Specification *of the mDA neuronal identity occurs within the proliferative zone (grey) of the ventral midline. Here, Msx1 and Foxa2 promote generic neurogenesis via regulation of Ngn2 whilst Lmx1a, supported by Msx1, specifies mDA neuron cell fate. As these mDA neuron progenitors become postmitotic and enter the intermediate zone (yellow), they begin to express the pan neuronal marker Tuj1 and, subsequently, the DA neuron transmitter regulator, Nurr1. Lmx1b and Wnt1 positively control early Pitx3 expression in some Nurr1^+ ^cells. The last stage in mDA neuronal *differentiation *proceeds as the Pitx3^+ ^cells and the Th^+ ^cells migrate ventrally into the peripheral zone (red). The ventrolaterally located Pitx3^+^Th^- ^cell subpopulations coalesce leaving a Pitx3^- ^Th^+ ^cell group in a medial position. Eventually, the early Pitx3 expressing subpopulation migrates laterally to make up the neural population of the SNc and begin to express Th. The remaining medial, Th^+^, cells form the VTA. All Nurr1^+ ^cells come to express Th and Pitx3. En1 and En2 maintain survival of the mature mDA neurons in the ventral midbrain. [Colours highlight the postulated region of activity of the genes in the corresponding colour.]

In the embryo, the pluripotent cells of the epiblast give rise to the multitude of different cell types of the adult animal. A complex series of extracellular signals and cell autonomous differentiation events transform these cells through germ layer specification, regionalisation and finally cell specific determination. In the generation of neurons of the SNc, research has focused on three major transitions: regionalisation of the neural plate, midbrain cell fate determination and finally terminal differentiation into mDA neurons (Figure 1). Differentiation of epiblast cells in culture into dopaminergic neurons parallels this pathway and knowledge gained from one system contributing to research in the other.

### Midbrain Regionalisation

Regionalisation begins early in neural plate development. At its inception the neural plate is axially defined: anterior-posterior and dorsal-ventral. The lateral edges of the neural plate roll up during neuralation becoming dorsal in the newly created tube. The neural plate midline becomes the ventral-most part of the developing nervous system. This positions the midline cells to receive extrinsic signals from the underlying mesoderm as well as signalling factors from within the neural plate (Figure 1 Regionalisation). During the establishment of regional identity the anterior neural tube develops a morphological constriction that co-localises with the boundary that defines and instructs cell fate restriction: the midbrain hindbrain border (MHB). The MHB, in conjunction with extrinsic signals from the mesoderm and endogenous regional patterning genes, specifies tightly localised populations of neural tissue in the flanking midbrain and hindbrain territories. The regional specification of the midbrain establishes a molecular identity that potentiates the cells of the ventral midbrain neuroepithelium to respond to subsequent dopaminergic fate determining signals.

The axial position of the MHB is established and maintained by Fgf8 expression and the co-repressive interactions of Otx2 and Gbx2 [8-11]. MHB signalling genes, Wnt1 and Engrailed 1 and Engrailed 2 (En1/2) are required for proliferation and survival of the ventral midbrain cells and Fgf8 is an instructive signal required for mDA neuron determination [3,12,13] (Figure 1 Regionalisation). In addition, *Wnt1 *mutant mice showed a loss of the mDA neuronal transcription factor *Pitx3 *expression in the Th^+ ^cell population. Although Fgf8 is not expressed in the midbrain, it clearly plays an extrinsic role in defining midbrain cell fate. Its ability to generate ectopic mDA neurons from naïve forebrain tissue has been shown and the blockage of FGF signalling results in a loss of mDA neurons [3,13]. Since Fgf8 can induce *Wnt1 *expression it is not clear if Fgf8 acts directly on mDA neuron progenitor populations or through induction of Wnt1 [14].

Along with Fgf8 and Wnt1, Shh and Otx2 are required for specification of the midbrain region [15-17]. Interference with Shh or Otx2 signalling results in malformation or absence of the midbrain. The midbrain of the *Shh *mutant is reduced in size, has extensive patterning defects and contains no *Th*^+ ^cells [18]. In the *Otx1/2 *double mutant, the entire head including the midbrain is absent. More informative for the study of midbrain development was the discovery that a subtle shifting of *Otx2*'s caudal expression boundary effects mDA neuron population size; a caudal shift results in an increase in population and a rostral one a reduction [19-21]. This increase or decrease is inversely reflected in a change in the seratonergic population, the ventral neuronal population of rostral hindbrain. These results demonstrate an Otx2 regulation of midbrain size but more importantly the switch from dopaminergic to seratonergic fate illustrates the change in regional identity controlled by Otx2 expression [22]. The ventral midbrain is also uniquely affected by Shh. Shh patterns ventral neuronal fate in the brain and the spinal cord but it is only ventral cells with the regional identity of the midbrain that respond to Shh expression by acquiring a dopaminergic cell fate [23-25].

In the spinal cord, the dorsal-ventral pattern of combinatorial and mutually exclusive areas of gene expression that determine the neural subtype is well established [26,27]. The pattern is initiated by Shh expression being induced in the floor plate cells, the ventral midline, by extrinsic Shh signalling from the underlying notochord. In the midbrain, this creates a signalling centre that, in combination with Otx2, sets up a regionally specific dorsoventral pattern of gene expression. Electroporation of *Shh *laterally, in the midbrain, induces a dorsally shifted archate pattern of expression of midbrain neuronal cell fate markers [28]. More dramatically a small point of ectopic *Shh *expression in the lateral midbrain generates a miniature series of bands of molecular markers duplicate to those that flank the endogenous midline [28].

Shh through binding its receptor, patched, derepresses smoothened to initiate a signalling cascade that eventually converges on the Gli family of transcription factors [15,29]. Dissection of the Shh signalling pathway in the midbrain points to a Gli2A-mediated control of floor plate Shh induction prior to E9 [23]. A second cascade initiated by Shh signalling represses ventral midbrain cell death resulting from cleavage of Gli3. The complete loss of Gli2A results in a severe reduction in mDA neurons and absence of *Isl1*^+ ^cells and *Nkx2.2*^+ ^cells, the more lateral ventral midbrain neuronal subtypes. The loss of *Gli2A *expression at E9 results in only a moderate decrease in the mDA neurons population but, again, a complete absence of the *Isl1*^+ ^and *Nkx2.2*^+ ^neurons [23]. By E11.5, neither the Gli2A-mediated Shh signalling nor the expression of Shh itself is required for normal development of the ventral midbrain neuron populations. [23,30] This suggests Shh expression in the midbrain neuroepithelium specifies the mDA neuron progenitor population shortly after Shh induction but more prolonged signalling is necessary to establish more lateral neuronal identity. Expression is restricted to the floor plate and patterning is affected in lateral tissue therefore this signalling acts over many cell diameters distance [15]. Shh signalling from the floor plate seems to generate a cascade of cell differentiation events requiring no further extrinsic instructive signals that result in the pattern of the neural phenotypes of the ventral midbrain.

Recent work with conditional mutants precisely targeted to dissect the spatial regulatory activities of Otx2 has greatly broadened our understanding of the gene interactions necessary to create the midbrain dorsoventral pattern of neuronal fates [17,31,32]. *Otx2 *is normally expressed in all midbrain cells. Driving *Otx2 *expression by the *Otx1 *regulatory region results in a loss of lateral *Otx2 *expression and a lateral expansion of the *Shh *expression region [32]. Normally, Shh is expressed only in the most ventral population, which give rise to the mDA neurons, flanked by a *Nkx6.1*^+ ^region, which generates the red nucleus (RN) progenitors, more lateral is the *Nkx2.2 *expressing region and the dorsal cells express *Pax3*. Coordinated with the induction of a broader *Shh *domain is a dorsal shift of the *Nkx6.1 *and *Nkx2.2 *expressing regions and a reduction of the *Pax3 *region. This change in the dorsoventral pattern leads to an increase in mDA neurons and a loss of RN neurons. A second mutant, regulating *Otx2 *expression using the *En1 *promoter, produces a loss of both ventral and lateral *Otx2 *expression [31]. The outcome of this manipulation was a similar lateral expansion of *Shh*, a dorsal shift in *Nkx6.1 *expression and a dorsal reduction of the *Pax *expressing region but in this case the *Nkx2.2 *expanded ventrally into the normal *Shh *expressing territory. In contrast to the previous result, the combined loss of ventral and lateral *Otx2 *midbrain expression causes a severe reduction in mDA neurons. These experiments indicate that endogenous lateral Otx2 expression maintains Shh in its ventral domain preventing the Shh inhibition of Nkx6.1. Additionally, Otx2 represses ventral expression of Nkx2.2, which can inhibit mDA neuronal differentiation

Fgf8, Shh and Otx2, genes involved in the early regional specification, are capable of promoting dopaminergic cell specification in greater abundance in their normal location as well as ectopically in naïve hindbrain or forebrain tissue [13,21,25,28,32]. The genes involved with subsequent determination, such as Lmx1a, also required for dopaminergic differentiation, are limited in their inductive powers to increasing the population of mDA neurons only in the midbrain [24]. This reflects the decreasing plasticity of the developing nervous tissue as it progresses through cell fate determination.

### Dopaminergic fate determination

The last few years have been rich in data contributing to understanding of the interactive molecular cascade involved in the multi-step differentiation of the mDA neuron progenitor population as well as identifying key players: Lmx1a, Ngn2, Fox2a, and Shh [23,24,29,30,33,34]. As the neuroepithelium of the midbrain develops, it thickens by cell proliferation and becomes layered. The cells in a narrow band adjacent to the ventricle retain their proliferative neural stem cell properties while other cells move out of this proliferative zone, exit the cell cycle and begin to differentiate. The mDA neuron progenitors inhabit the ventral most proliferative zone and move ventrally and then laterally as they differentiate (Figure 1 Specification). These cells express Lmx1a, Foxa2, Lmx1b, Msx1/2, Ngn2 and, at early stages, Shh. Cells at this stage acquire mDA neuronal specification and begin the final steps of postmitotic differentiation. They begin to express Nurr1 as well as the mDA neuron specific molecule, Pitx3, in the intermediate zone, which is ventral to the proliferative zone. As they reach the ventral peripheral edge of the neuroepithelium, they are fully differentiated mDA neurons and express Th (Figure 1 Differentiation) [5].

The genes expressed in the ventral proliferative zone have highly interactive regulatory mechanisms. Lmx1a, Foxa2, Ngn2, Lmx1b, Msx1/2, and Shh have all been shown to be necessary for normal mDA neuron development but of them only Shh and Lmx1a are both necessary and sufficient to induce mDA neurons [24]. The data on Shh participation in midbrain patterning has been discussed in the previous section. Two different studies have indicated that Shh is required only prior to E10.5 in mDA neuron determination and mDA neuron progenitor cells continue to be born after *Shh *is downregulated in the ventral midbrain [23,30]. Lmx1a, on the other hand, is continually expressed during mDA neuron progenitor production [35]. Silencing *Lmx1a *in the developing midbrain with siRNA results in a dramatic reduction in *Nurr1*^+ ^cells whilst its ectopic expression results in an expansion of the *Nurr1 *expressing midbrain region [24]. These data, in combination with Shh's ability to induce firstly Lmx1a and subsequently Th in naïve midbrain explants, indicates that Shh promotes production of mDA neurons through induction of Lmx1a [24]. Shh also is capable of regulating *Foxa2 *expression which in turn positively regulates *Ngn2 *expression (Figure 1)[30,32]. Interestingly, *Foxa2 *is required for normal Nurr1 expression from E10.5, the end point of Shh participation [30]. Ngn2 is also upregulated by Msx1/2 which can be induced by Lmx1a overexpression in midbrain tissue [24].

The transition of a cell from the proliferative zone to the intermediate zone is mediated by Ngn2. Loss of function mutation of *Ngn2 *dramatically delays and reduces the number of Nurr1^+ ^cells in the intermediate zone and does so in a dose dependent manner [32-34]. Compound deletion of the *Ngn1 *and *Ngn2 *alleles increases the severity of this reduction [30,34]. The involvement of Ngn2 in neurogenesis is highlighted by the decrease in both total number of cells and number of neurons in the mutant intermediate zone [30,33,34]. The decrease of cell number is not associated with apoptosis but instead with a reduction in *Hes5 *and *Dll1*expression, markers of proneural activity the ventricular zone [34]. Therefore, the cell loss is likely to be the result of a retardation of the rate of ventricular zone to intermediate zone transition. In the *Ngn2 *mutants, depleted Nurr1^+ ^population do express Th and there is some recovery of the Nurr1^+ ^population during development. This presents the possibility of an Ngn2-independent pathway of mDA neuron differentiation. There is evidence that in the mutant state Mash1 can compensate for the loss of Ngn2 but since the deletion of *Mash1 *in normal embryos has no effect on mDA neuron development it is probable that Ngn2 is the normal mediator of neurogenesis [34]. Using Ngn2-GFP mice, Thompson et al were able to demonstrate that all mDA neurons were derived from Ngn2-GFP^+ ^cells both in culture and in the recipient striatum following transplantation [36].

Msx1 is also expressed in the proliferative zone. While Msx1 can not induce Lmxla, it does act synergistically with Lmx1a to promote the mDA neuron progenitor population through inhibition of Nkx6.1 on the lateral edges of the ventral proliferative zone [24]. This inhibition results in Ngn2 expression in those areas. In the absence of Lmx1a, the Msx1-mediated Ngn2 expression does not result in the induction of Nurr1 [24]. This demonstrates the need for pre-specification of the Ngn2^+ ^proliferating cells by Lmx1a to determine the mDA neuron phenotype (Figure 1 Specification).

### Terminal differentiation

The final step in the differentiation of dopaminergic neurons is the expression of Th, the rate limiting enzyme in the production of dopamine. Th expression is not initiated in mDA neurons of *Nurr1 *null embryos [37-39]. However, the mDA neuronal lineage is properly specified as demonstrated by the expression Pitx3 and Aldh1a1 at the appropriate stages in the ventral midbrain of *Nurr1 *mutant mice. Therefore, Nurr1 controls the dopaminergic neuron transmitter phenotype but does not play a role in the specification of mDA neurons. Recently it has been shown that Nurr1 regulates *Th *by activating the NGF-1 response element in the *Th *promoter region after its own phosphorylation by ERK1/2 [40,41]. Furthermore, the onset of Parkinson's disease parallels the interruption of this interaction [40].

Another transcription factor implicated in the regulation of Th and the proper differentiation of a subset of mDA neurons is the paired-like homeodomain protein Pitx3. Pitx3 is expressed in both the SNc and the VTA DA neurons [42,43]. However, lack of Pitx3 results in the preferential loss of the SNc neuronal sub-population whereas the VTA neurons are relatively intact, a phenotype that closely resemble that of Parkinson's disease [44-47]. Furthermore, analysis of the Pitx3-GFP knockin mice revealed a restricted down-regulation of Th in the SNc cells prior to their death [47]. The preferential cell loss is closely linked with the segregation of Pitx3 expression in two apparently discrete mDA neuron precursor cell populations during the onset of terminal mDA neuron differentiation. Cells located at the ventrolateral position of the marginal zone express Pitx3 prior to Th whilst cells positioned dorsomedially express Th before Pitx3 [47]. It is thought that the ventrolateral cells go on to form the SNc whilst those more medial become the VTA. The differential regulation of Pitx3 in SNc dopaminergic precursor cells dictates their strict dependence on Pitx3 for survival. The heterogeneity of the mDA neuron population and their differential dependence on Pitx3 has been further demonstrated by the identification of *Aldh1a1*, a retinoic acid synthesising enzyme, as a SNc specific Pitx3 target gene and the partial rescue of *Pitx3 *deficient phenotype by retinoic acid [48].

Finally, the En1 and En2 homeobox transcription factors are required for the maintenance of the mDA neurons in late stages of foetal development and in the adulthood. In the *En1/2 *double mutants all mDA neurons die between E12, the onset of En1 expression, and E14 [49]. While the *En1*^+/-^; *En2*^-/- ^mutant has the normal mDA neuronal compliment at birth, it then begins to loose Th^+ ^neurons in the SNc while retaining those of the VTA [50]. These results indicate that En1 is required for mDA neuron maintenance and, more specifically, highlight the distinct survival requirements of SNc and VTA neurons.

## Steering ES cell differentiation into a DA fate

It is generally accepted that much of ES cell differentiation in vitro mimics vertebrate development as described above. Therefore, induction of a specific cell fate should involve the application of known extracellular factors in a stepwise manner during the course of ES cell differentiation. The aim is to induce a cascade of transcription resembling normal development. Indeed, most differentiation paradigms reported to date apply Shh and FGF8 to differentiating ES cell cultures [51-57]. A panel of survival-promoting factors including glial cell line-derived neurotrophic factor, neurturin, transforming growth factor-β_3 _and dibutyryl-cAMP have been shown to promote the maturation and survival of ES cell-derived Th^+ ^neurons [58]. Furthermore, foetal midbrain astrocytes can promote the production of DA neurons from ES cells [59].

Induction with Shh and FGF8 during ES cell differentiation can result in a culture in which ~30% of the neuronal population expresses Th. In these studies Shh and FGF8 are applied at the time when neural progenitor production (ie. Nestin^+^, Sox1^+ ^cells) is at its peak. This is based on the understanding, supported by developmental studies, that Shh induces a DA fate via acting on ES cell derived Nestin^+ ^Sox1^+ ^neural progenitors. However, we found that Shh and FGF8 were unable to stimulate more DA neurons in FACS (fluorescence activated cell sorting) purified Sox1^+ ^neural progenitors than observed in un-sorted control cultures [60]. This finding indicates that DA induction occurred in 'primitive' neural progenitors prior to or at the point of Sox1 expression in differentiating ES cells.

DA neurons can also be efficiently induced by stromal cell-derived inducing activity (SDIA) produced by bone marrow derived PA6 stromal cells [61]. This protocol, which involves culturing ES cells on a layer of PA6 cells, can generate a high proportion of Th-positive neurons comparable to those derived through Shh induction as discussed above. Although the nature of SDIA remains unclear, it was suggested that SDIA might be a secreted factor that is secondarily tethered to the cell surface. A recent study implicates Wnt5 as a component of SDIA [62]. Co-culture of FACS purified Sox1^+ ^neural progenitors with PA6 did not yield proportionally more dopaminergic neurons as compared to cultures on poly-lysine and laminin, suggesting that SDIA mediated DA induction also occurs at pre-Sox1 expression stage [60]. However, SDIA has a generic promoting effect on the proliferation and survival of foetal and ES cell-derived neural progenitors ([63], authors unpublished observation).

To date, the most efficient production of dopaminergic neurons from ES cells is achieved via genetic manipulation of transcription factors that are normally active in midbrain DA neurons [24,47,64-67]. Nurr1 is able to induce Th expression in central nervous system precursors derived from cortex, midbrain tissue, the lateral ganglionic eminence and from ES cells [46,64,67-71]. In Nurr1 expressing cultures, enhanced production of Th-positive neurons was associated with an increase in the expression level of AADC, DAT, VMAT, and Pitx3. However, engineered Nurr1 also induces Th expression in ES cell derived non-neuronal progeny [72]. This indicates that, consistent with mouse genetic studies, Nurr1 controls Th expression independent of their neuronal cell fate specification [37].

## Regional (midbrain) identity of ES cell-derived DA neurons

The pathological hallmark of Parkinson's disease is the preferential loss of the substantia nigra subgroup of mDA neurons. Previous transplantation studies demonstrated that only midbrain dopaminergic cells are therapeutically useful in cell replacement therapy in animal models of Parkinson's disease [73]. Furthermore, transplanted DA neurons that form synaptic connections with the host striatum exhibit characteristics of the substantia nigra neurons demonstrated by morphology and the expression of Girk2 (a marker that is preferentially expressed by adult nigral neurons) [36]. Therefore, cells to be developed with Parkinson's disease therapy in mind must possess the capacity to generate mDA neurons, ideally those of substantia nigral character.

With increasing recognition of the necessity of generating DA neurons of 'true' midbrain property, many recent studies have investigated the induction, in ES cell derived neuronal cultures, of midbrain neural progenitor markers (e.g. Pax2, En1, Lmx1a) and the generation of midbrain specific post mitotic DA neurons (Pitx3^+^, Aldh1^+^) either by RT-PCR or immunocytochemistry [24,43,65,74-76]. The homeobox protein Pitx3 is the most specific mDA neuronal marker known to date, due to its exclusive expression in mDA neurons and their postmitotic precursors [42,43]. The authors have previously generated Pitx3-GFP knock-in ES cells as a tool for tracking mDA neuron differentiation in vitro [43]. ES cell derived Pitx3-GFP^+ ^neurons co-express almost completely with known midbrain DA markers including Pitx3, engrailed, Lmx1a, Raldh1 (Ahd2), Nurr1 and pan-DA neuron markers such as AADC, VMAT2 and Th [43,47,77]. However, only ~15% of SDIA induced ES cell-derived Th^+^DAT^+ ^neurons were Pitx3-GFP^+^, demonstrating that ES cell-derived DA neurons are heterogeneous and do not all exhibit midbrain identity [43]. Similarly, human ES cells also generate DA neurons of different regional identity [78]. More recent work with mouse ES cells showed a lack of mDA specific neuronal marker expression in Th^+ ^neurons derived using a monolayer differentiation protocol [24,60,79] whilst an embryoid body based protocol gave rise to cultures with a high proportion of Th^+ ^neurons expressing Pitx3-GFP [77]. Shh was applied in both the monolayer and embryoid body differentiation paradigms so the varied efficiency in generating midbrain-specific DA neurons does not seem to be due to limited Shh signalling. On the other hand, PA6-co-culture and embryoid body based ES cell differentiation protocols share a common feature: high local cell density at neural fate transition stage. In contrast, efficient monolayer neural differentiation entails low cell density. Together, these studies suggest that close cell-cell interaction is necessary for induction of mDA neuron phenotype.

Consistent with its role in mDA neuron ontogeny, expression of the Pitx3 transgene in mouse ES cells can promote the generation of Th^+^Nurr1^+^DA neurons co-expressing a panel of midbrain markers such as En1, Ahd2 and endogenous Pitx3. In contrast to Nurr1 overexpression, Pitx3 manipulation does not result in a significant increase in the overall number of Th^+ ^cells [47,65]. These findings reinforce the concept of parallel transcription machinery for DA neural transmitter phenotype and for midbrain DA neuronal identity [80]. Indeed the combined transduction of Pitx3 and Nurr1 virus lead to an increase in *Th *and *Dat *RNA expression in neural differentiated human ES cells [66]. However, a synergistic effect between these two factors on Th expression was not observed in mouse ES cells in the same study. Recently, Lmx1a and Msx1 have been shown as potent stimulators of Pitx3^+^En1^+ ^DA neuron generation from mouse ES cells [24]. The effect of Lmx1a requires prior Shh potentiation so exogenous supply of Shh during ES cell differentiation is needed. Msx1 can only induce Th^+ ^neuron production when it is co-transfected with an Lmx1a vector. However, transduction of Lmx1a, Msx1 and/or Pitx3 virus in cultured rat mesencephalic neural progenitors, alone or combined, did not increase the number of Th^+ ^cells [81]. Interestingly, another study observed an enhancement of Th^+ ^neuron differentiation following the co-culture of Pitx3 transfected rat neurosphere neural progenitors with foetal ventral mesencephalic tissues [82]. Together these studies suggest that the intact ventral mesencephalic tissue and differentiating ES cell cultures contain additional instructive molecules and/or exhibit intrinsic characteristics necessary for dopaminergic neuron determination.

## Making DA neurons from neural stem cells

Although all available evidence demonstrates that ES cells have a greater capacity to produce dopaminergic neurons than neural stem cells (NSC) or other types of lineage-specific stem cells [52,83], for cell therapy, it may be preferable to derive DA neurons from neural stem cells (NSC). The advantages of using NSC over ES cells are two fold. Firstly, as a cell type already restricted to the neural lineage, NSC do not generate teratoma, a multilineage tumour originating from residual undifferentiated ES cells following transplantation [69,84]. The formation of teratomas in ES cell transplant is not only unacceptable for clinical applications, these tumour formations significantly compromise the health of the recipients and thus has hindered long term follow up behaviour analysis of grafted animals. Secondly, NSC can be derived from foetal and adult brains, thus one could envisage the potential use of a patient's own NSC in cell therapy to avoid immune rejection.

NSC can be routinely established by propagating foetal or adult neural tissues and ES cell derived neural progenitors in chemically defined culture media in the presence of FGF and EGF. However, whether propagated adherently or as cell aggregates (neurospheres), FGF and EGF expanded NSC primarily give rise to GABAergic neurons with no apparent regional identity [85-87]. This may be attributed to the deregulated dorsoventral patterning of neural progenitors by FGF during in vitro expansion [88]. The potential of NSC as a therapeutic reagent relies on our ability to maintain its full neurogenic competence. Using the human ES cell differentiation model, two recent studies identified a primitive NSC stage from which a broader spectrum of neuronal subtypes (including dopaminergic neurons) can be produced [76,89]. These primitive NSC are present at very early stage of ES cell neuralisation prior to FGF/EGF expansion and express similar molecular markers with that of early anterior neural epithelial cells. Morphologically, these primitive NSC assemble themselves as neural rosette and/or neural tube structures and were indeed termed as rosette neural stem cells (referred as R-NSC) by Elkabetz et al., [76]. These authors showed that Shh and Notch signalling, together with a high density culture environment, can prolong the maintenance of the rosette morphology. In contrast, FGF and EGF maintained NSC do not display rosette features and show a greater preponderance of GABAergic neuronal fate. Future work should address whether the maintenance of neural rosette morphology by Notch and Shh indeed is coupled with the maintenance of the neurogenic potency of R-NSC.

Elucidation of the transcription programme associated with R-NSC status may lead to the discovery of master regulators governing NSC potency. A list of candidate marker genes has been identified by transcriptome profiling for the primitive NSC and R-NSC [76,89]. Regulated expression of some of these molecules may be key to preserving the full neurogenic repertoire of NSC. The identification of primitive NSC master regulators may offer the means to re-programme FGF/EGF expanded NSC back to a stage resembling early anterior neuroepithelium, in a fashion analogous to the generation of induced pluripotent stem (iPS) cells.

A similar approach may be taken to program dopaminergic competent NSC by exploiting transcription factors known to act in this specific neuronal lineage. For example, combined gene manipulation of Nurr1 (controlling dopaminergic neurotransmitter phenotype) with Lmx1a or components of the Wnt/Lmx1b-Pitx3 pathway (midbrain identity, Figure 1) might be able to impose a midbrain DA potential in FGF/EGF expanded NSC.

One should note that for clinical consideration, it is desirable to produce the therapeutically important somatic cell types using extrinsic growth factors or small molecules during the course of ES cell in vitro differentiation. While we are progressing toward discovering new soluble reagents, genetic based programming provides an important tool to decipher the pathways and mechanisms controlling stem cell fate choice which is fundamental for the establishment of culture parameters capable of sustaining the full complement of NSC potential. Furthermore, the timely investment in iPS related studies should prompt rapid technological developments such as protein transduction or small molecule mediated gene regulation.

## Summary

The past few years have seen a rapid progress in our understanding in the genetic control of mDA neuron fate specification during foetal development. Combinatorial expression of transcription regulators provides markers for ventral mesencephalic neuroepithelial cells of different regional zones and/or at different developmental stages. These mDA neuron lineage markers have greatly facilitated the assessment of 'authentic' mDA neural phenotype that can be derived from ES cells. In addition, the availability of defined mDA neuron progenitor populations with distinct differentiation capacity isolated by lineage marker expression from existing reporter mice and ES cells has initiated the search for the most suitable cell population for cell-based transplantation therapy [77,90]. Most importantly, knowledge of intrinsic and extrinsic mDA neuron developmental cues has been successfully translated into novel strategies for mDA neuron production in vitro from ES cells. Combining enhanced efficiency of generating mDA neurons in vitro with the use of reporter ES cell lines that track mDA neuron precursors of different developmental stages, the ES cell based differentiation assays offer a simplified model system in which to dissect the interactions of intrinsic and extrinsic signals controlling mDA neuron specification. DA neurons generated from iPS cells, especially those harbouring Parkinson's disease mutations, provide a potential tool to study the disease process and to test the efficacy of preventative or therapeutic drugs. Even though great progress has been made, our understanding of the genetic cues controlling the induction of the mDA neuron progenitors and the specification of the mDA neuronal fate is far from complete. Further advances in the field will undoubtedly facilitate the controlled differentiation of pluripotent stem cells into clinically relevant DA neurons.

## Competing interests

The authors declare that they have no competing interests

## Authors' contributions

All authors participated in developing the ideas, the writing, discussion and integration of information. All authors read and approved the final manuscript.
